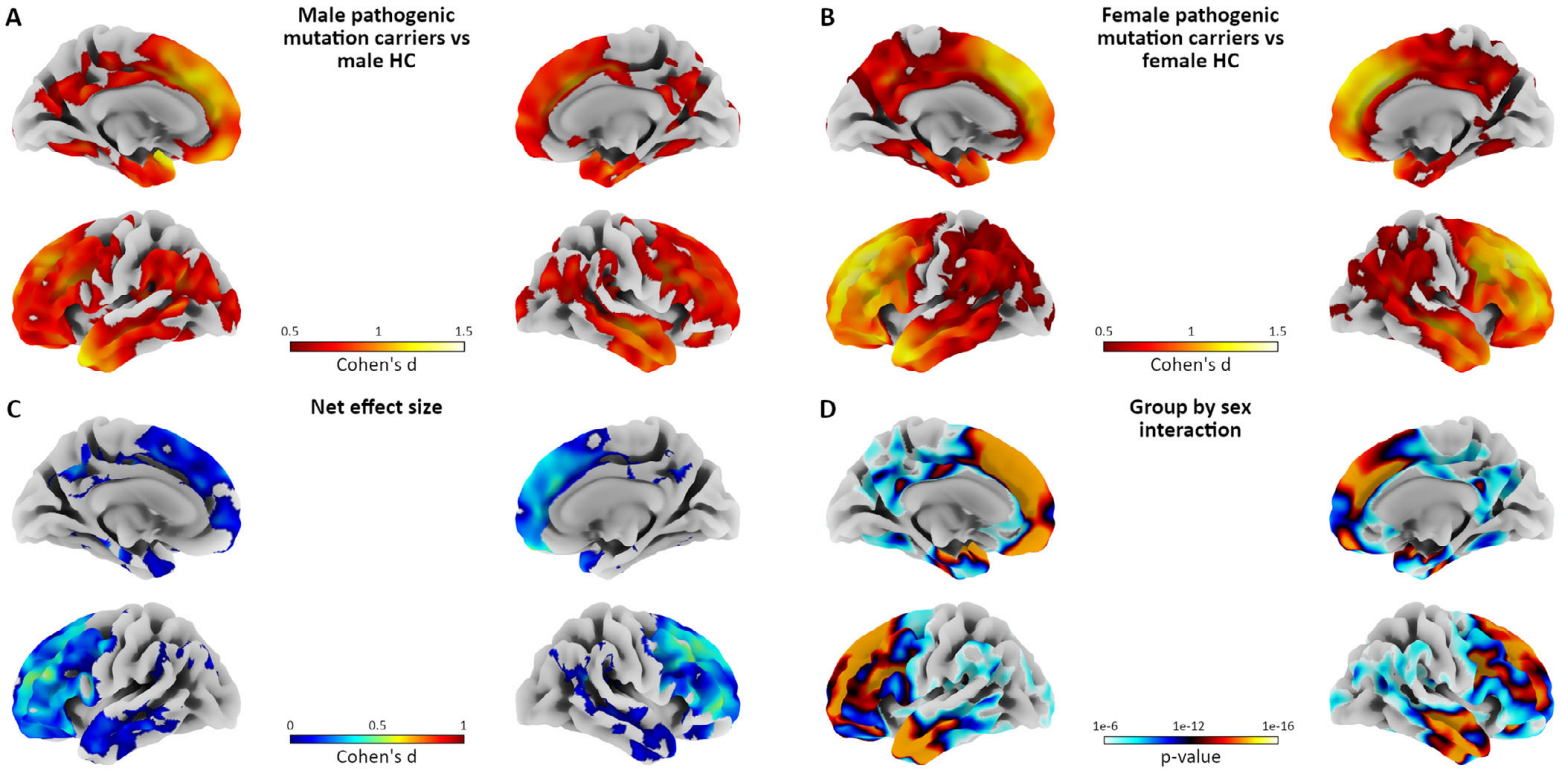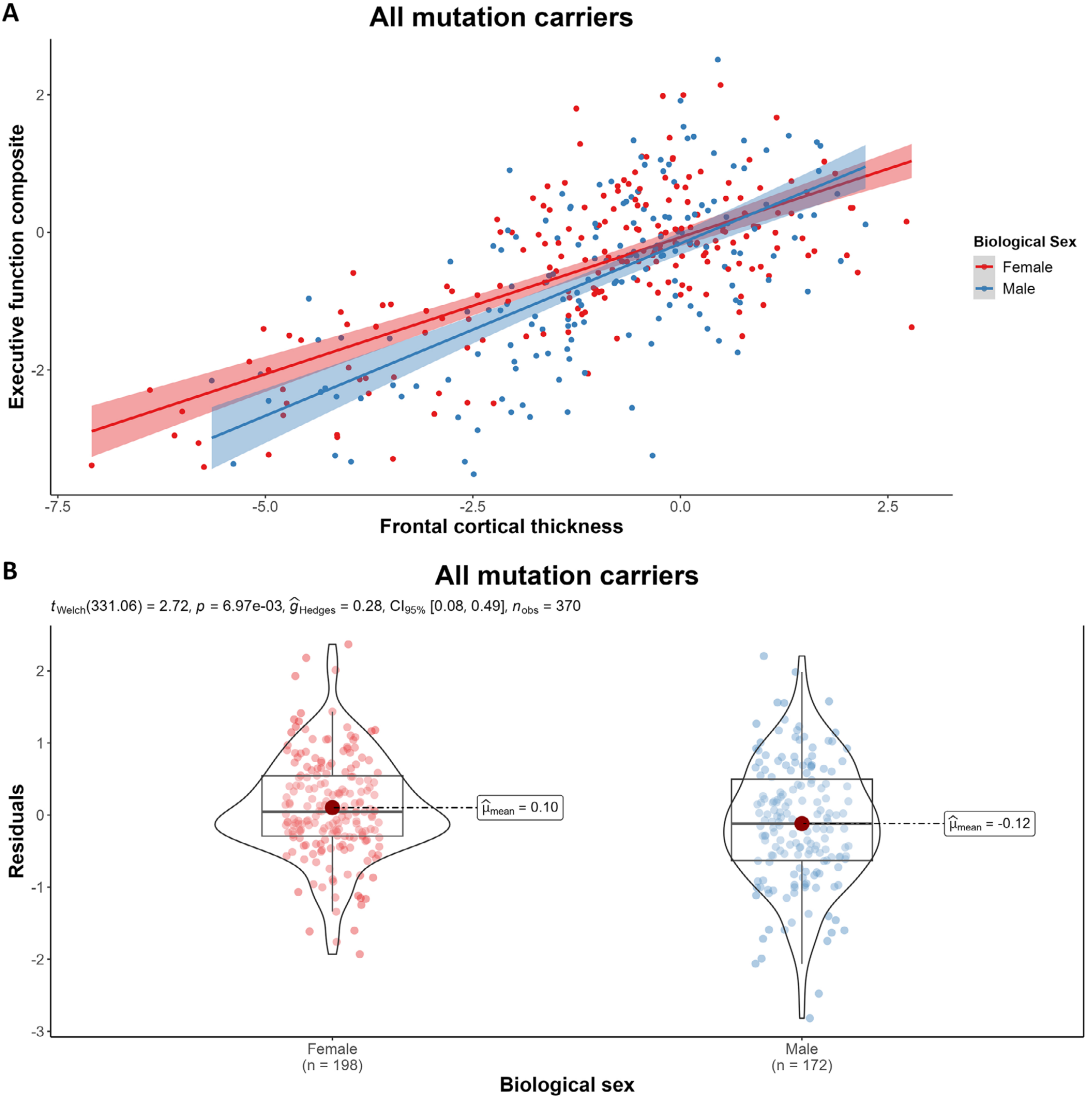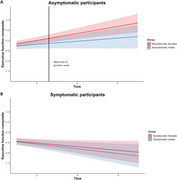# Behavioral and executive reserve as measured with MRI in women with autosomal dominant FTD

**DOI:** 10.1002/alz70856_102565

**Published:** 2025-12-25

**Authors:** Jesús Garcia Castro

**Affiliations:** ^1^ Sant Pau Memory Unit, Hospital de la Santa Creu i Sant Pau ‐ Biomedical Research Institute Sant Pau ‐ Universitat Autònoma de Barcelona, Barcelona, Barcelona, Spain

## Abstract

**Background:**

Biological sex impacts resistance to neurodegeneration, yet its role in genetic frontotemporal dementia (FTD) remains uncertain.

**Method:**

We examined 394 genetic FTD patients and 279 controls from the ALLFTD consortium, evaluating annual neuropsychological performance and MRI‐derived cortical thickness. Clinical characteristics and cortical thickness were compared between sexes. Cognitive measures and cortical thickness values were normalized against sex‐matched controls. Residuals from linear regression models served as proxies for cognitive reserve, and sex differences in longitudinal trajectories were analyzed using linear mixed‐effects models.

**Results:**

There were no differences in clinical or demographic variables, nor in cognitive function and behavior outcomes between sexes. Symptomatic females with genetic FTD exhibited lower frontal cortical thickness than males. In the *C9orf72* subgroup, females showed lower‐than‐expected frontal cortical thickness relative to their executive function levels. Sex‐related differences in cognitive reserve were most pronounced near symptom onset but diminished over time.

**Conclusion:**

Females with genetic FTD demonstrated greater cognitive reserve than males, indicating that biological sex influences resilience to frontotemporal neurodegeneration.